# Pooled RNAi Screens - Technical and Biological Aspects

**DOI:** 10.2174/138920210791110988

**Published:** 2010-05

**Authors:** M. Boettcher, J.D. Hoheisel

**Affiliations:** Division of Functional Genome Analysis, Deutsches Krebsforschungszentrum, Im Neuenheimer Feld 580, 69120 Heidelberg, Germany

**Keywords:** Pooled RNAi screen, molecular tag, barcode, shRNA library, microarray, half hairpin.

## Abstract

RNA interference (RNAi) screens have recently emerged as an exciting new tool for studying gene function in mammalian cells. In order to facilitate those studies, short hairpin RNA (shRNA) expression libraries covering the entire human transcriptome have become commercially available. To make use of the full potential of such large-scale shRNA libraries, microarray-based methods have been developed to analyze complex pooled RNAi screens. In terms of microarray analysis, different strategies have been pursued by different research groups, largely influenced by the employed shRNA library. In this review, we compare the three major shRNA expression libraries with a focus on their suitability for a microarray-based analysis of pooled screens. We analyze and compare approaches previously used to perform pooled RNAi screens and point out their advantages as well as limitations.

## INTRODUCTION

RNA interference has become a popular tool for the analysis of gene function in model organisms like *C. elegans* and *D. melanogaster *[1-8]. In 2001, Elbashir *et al. *[9] discovered that RNAi also suppresses gene expression in mammalian cells. Since then, loss-of-function studies, commonly performed by transfection of short interfering RNAs (siRNAs), have greatly facilitated functional analysis of the human transcriptome [10-12]. However, there are major downsides to siRNA experiments, most importantly the transient inhibition of gene expression, as well as their inefficient transfection into non-dividing cells. In order to overcome those limitations, several groups developed short hairpin RNA (shRNA) expression vectors, which can be stably integrated into a target cells genome *via *retro- or lenti-viral gene transfer [13-17]. Intracellular processing of shRNAs results in short duplex RNAs with siRNA-like properties [18, 19]. Viral integration ensures not only a broad range of infectable target cell types, but also the stable expression of specific shRNAs, resulting in the permanent reduction of the targeted gene product. Three different research groups have created shRNA expression libraries, which have recently become commercially available [15-17]. Each of those libraries allows the targeted knockdown of thousands of different genes, thus greatly facilitating functional analysis of the whole transcriptome through loss-of-function studies.

## POOLED RNAi SCREENS

In addition to the stable knockdown of individual genes in an arrayed format, large-scale shRNA expression vector libraries render another exciting application possible, namely pooled RNAi screens. A number of pooled screens have been performed so far, with viral pools containing up to 45,000 different shRNA expression vector constructs [16, 20-27]. All of those screens were based on the principle of integrating not more than one shRNA expression vector per target cell. Consequently, only one specific gene is targeted for knockdown in each resulting clone. Moreover, the genomically integrated shRNA template sequence serves as a molecular tag, providing information about the identity of the expression vector harbored in each cell. An early way to make use of molecular tagging in order to analyze pooled screens was to simply pick individual colonies that survived a positive selection screen followed by sequencing of their tag [16]. Since this approach is extremely time consuming, Brummelkamp *et al.* introduced a concept termed ‘siRNA bar-code screen’ [28] similar to pioneering studies in yeast [29, 30]. This DNA microarray-based method employs PCR-amplified shRNA template sequence pools from a test as well as a reference condition (Fig. (**1**)). Each PCR fragment pool is either labeled with a different fluorophore, followed by hybridization of both pools to the same DNA microarray, or labeled with the same fluorophore and hybridized to individual microarrays. Immobilized on the microarray surface are single-stranded DNA sequences complementary to the PCR-amplified shRNA template sequences. After hybridization, the signal intensity ratio between both conditions is determined for each probe sequence. Ratios reflect the relative abundance of cells expressing a certain shRNA under test conditions as compared to the reference. Consequently, constructs expressing shRNAs that sensitize cells to the applied selective conditions will be depleted from the pool, whereas constructs rendering cells resistant will be enriched (Fig. (**1**)).

Berns e*t al.* demonstrated the feasibility of the analysis of pooled RNAi screens in mammalian cells by means of ‘siRNA bar-code screens’ [16]. The shRNA library they used, termed the NKI library, is one of three commercially available libraries. The other two were designed by the groups of Hannon and Elledge (H&E library [15]) and The RNAi Consortium (TRC library [17]), respectively. Selected features from all three libraries are summarized in Table **1**. Researchers can order from the indicated suppliers individual constructs as well as subsets of constructs targeting whole gene families. One of the most noticeable differences between the three libraries is certainly their coverage, with the H&E library targeting the expression of the highest number of human genes (18,000) followed by the TRC library (15,000) and the less complex NKI library (8,000). However, a high redundancy of the library is also important in order to reduce false positive results that are due to off target effects. In that respect, the TRC library is unmatched with an average coverage of five shRNA expression constructs for each of the targeted 15,000 genes.

## KNOCKDOWN EFFICIENCY

When talking about RNAi, it is impossible not to talk about knockdown efficiency. As a rule of thumb, at least one out of three shRNA expression constructs targeting a certain gene is generally promised by the suppliers to reduce gene expression by at least 70%. A major concern when making such statements, however, is commonly neglected, namely the large variations in knockdown efficiencies between different cell lines. This issue is most strikingly illustrated by a data set provided on the website from Open Biosystems [31]. It shows the residual target gene expression of 132 cancer genes in the ovarian carcinoma cell line OVCAR-8, as well as the breast carcinoma cell line MCF-7. In total, 393 pGIPZ constructs from the H&E library were introduced into both cell lines. While in OVCAR-8, almost every second construct (47%) succeeded to reduce target gene expression by more than 70%, in MCF-7 only every fifth shRNA expression construct did (19%). This data not only emphasizes the importance of careful target cell line selection, but also points out a major challenge in validating knockdown efficiencies for shRNA expression constructs. Certainly a step in the right direction is the TRC2 approach taken by the TRC together with Sigma-Aldrich, who are aiming at determining knockdown efficiencies for constructs present in their library on multiple cell lines. Their efforts have already resulted in the successful validation of shRNA constructs targeting the expression of more than 4,500 different genes. Utilizing those constructs for the assembly of validated high-efficiency shRNA expression pools could facilitate the parallel study of a large number of genes combined with minimized pool complexity.

## MOLECULAR TAGGING AND MICROARRAY ANALYSIS OF POOLED SCREENS

In order to decode pooled RNAi screens, molecular tags need to be PCR amplified, labeled and hybridized to DNA microarrays containing their complementary probe sequences (Fig. (**1**)). Thus, the nature of such tags is of great importance for the accurate analysis of pooled RNAi screens. Three different types of molecular tags have previously been used by different research groups, namely full-length hairpin template sequences, half hairpin template sequences and external barcode sequences located downstream from each shRNA template. Advantages as well as limitations of each type of tag are discussed in the following paragraphs.

### Full-Length Hairpin Tags

A number of studies employ the full-length shRNA template sequence as a molecular tag [16, 20, 21, 26]. A major challenge when working with full-length hairpin sequences however is their equal recovery *via *PCR. Due to the fact that shRNA sequences are typically around 20 nucleotides (nt) in length, self-annealing happens in a similar temperature range as annealing of the PCR recovery primers. The different sequence composition of each individual shRNA consequently leads to self-annealing of different constructs at dissimilar temperatures, resulting in unequal PCR amplification.

A second issue when employing full-length hairpin sequences as molecular tags is restrictions in labeling approaches. Since some labeling strategies, as for instance random priming, involve enzymatic reactions at 37°C, self-annealed sequences would prevent efficient labeling. A third problem with amplified shRNA template sequences occurs during hybridization of labeled PCR fragments to the DNA microarray. Self-annealed shRNA sequences are hindered in the hybridization to complementary probes on the array surface, resulting in low or absent signal intensities. This last issue is illustrated by findings from Schlabach *et al.* [24]. They analyzed a pool of 8,000 shRNA expression constructs *via *their full-length hairpin sequences and found that less than 0.8% of the probes generated signals greater than 2-fold the background intensity.

### Half Hairpin Tags

In order to circumvent the described problems with labeling and hybridization, different approaches have been taken by several groups to use only half the hairpin’s sequence as molecular tag. By placing the forward primer into the common 19 nt loop sequence of the H&E library, only the antisense half of the hairpin can be amplified and subsequent effects resulting from self-annealing can be avoided [23, 24]. A different approach is taken by researchers working with the TRC library [25]. Since the common loop sequence in the TRC library consists of only 6 nt, PCR amplification of half the hairpin’s sequence is not an option. Instead, half hairpins are generated following PCR amplification of the full-length hairpin sequence *via *restriction digest with the enzyme XhoI recognizing the 6 nt hairpin sequence. When analyzing a pool of 4,000 shRNA expression constructs *via *their half hairpin sequences, Schlabach *et al.* [24] found more than 72.2% of the probes generated signals greater than 2-fold the background intensity. Compared to the 0.8% of probe signals obtained from full-length hairpin tag analysis, this is a tremendous increase, indicating the strong impact of self-annealed target sequences on microarray hybridization. However, despite improved hybridization efficiency achieved *via *half hairpin analysis, secondary structures of the shRNA template sequence continue to interfere with PCR amplification of the tags.

Another important point concerning the use of half hairpin sequences as molecular tags is appropriate microarray probe design. Half hairpin sequences in any of the three available libraries consist of approximately 20 nt, limiting flexibility for probe design [23-25]. The diverse sequence composition of shRNA sequences in a pool consequently results in dissimilar hybridization properties for each probe. Or in other words, optimized hybridization conditions that work for one fraction of the probes on the microarray might be suboptimal for another fraction of probes. Although there have been attempts to equalize hybridization properties by adding parts of the common vector backbone to the probe sequences [24], options are limited.

### External Barcode Tags

An exclusive feature of the H&E library, which cannot be found in the NKI or TRC library, is the unique 60 nt long external barcode sequence present in every shRNA expression vector. Each individual shRNA template sequence is associated with a different external barcode sequence, allowing the identification of the encoded shRNA *via *its barcode [32]. All barcode tags are located downstream from the shRNA templates and were sequence-validated for each shRNA expression construct. Due to their non-complementary nature, external barcodes help to avoid any of the above mentioned full-length or half hairpin template associated problems. Since self-annealing is not an issue, neither PCR amplification nor labeling or microarray hybridization is hindered in any way.

Further, probe sequences for microarray analysis can be designed according to technical needs. Although previously 50 nt long probe sequences complementary to almost the full length 60 nt barcode were used [23], probe lengths can theoretically range from 15 up to 60 nt. This allows the straightforward design of probe sequences to obtain equally optimal hybridization properties for each probe sequence present on the microarray. The described advantages of external barcode tags were recently employed by our group to decode pooled RNAi screens [33]. By using six overlapping 25 nt long tiling probes to detect each 60 nt external barcode, we were able to precisely predict the abundance of individual shRNA expression constructs from a pool.

Taken together, external barcode sequence analysis displays a number of advantages over full-length or half hairpin analysis, most notably equal PCR amplification and flexible probe design. Probably the only disadvantage of the external barcode approach is that extensive sequencing of every new library is necessary to generate barcode information and linkage to specific shRNA sequences [24].

## POOL COMPLEXITY

Another important issue concerning hybridization of complex sample pools to DNA microarrays is unspecific probe-target interaction. This effect, commonly referred to as cross-hybridization, has been shown by Wick *et al.* [34] to increase with an increased complexity of the sample pool. Ever since the first pooled RNAi screen in mammalian cells [16], there has been a tendency to increase the complexity of employed shRNA pool sizes. Higher density oligomer microarrays further facilitated the analysis of increasingly complex shRNA pools. While initial studies used shRNA expression vector pool sizes of approximately 1,000 constructs [16], more recent publications employed pools containing up to 45,000 constructs [25]. Thereby the examination of effects caused by cross-hybridization has not received much attention. To our knowledge, only one study has addressed this problem so far [24]. By hybridizing 4,000 half hairpin sequences to a microarray containing the 4,000 complementary probe sequences, plus another 4,000 non-complementary ones, the authors could demonstrate that only 0.5% of 4,000 non-complementary probes showed a signal intensity higher than 2-fold the background. This goes to show that shRNA expression pools the size of up to 4,000 constructs can be analyzed by means of microarray technology without major unspecific probe-target interaction. Providers of the H&E as well as the TRC library offer not only individual constructs from their libraries for sale, but also predefined, gene family specific sub-libraries. These sub-libraries generally contain between 500 and 2,000 different shRNA expression constructs. Hence, according to Schlabach *et al.* [24], they can be analyzed by means of microarray hybridization without causing artifacts by cross-hybridization.

## IMPLICATIONS OF DEEP SEQUENCING ON POOLED RNAI SCREENS

With the recent emergence of widely available deep sequencing technology, a whole new approach for the analysis of pooled RNAi screens has become conceivable. As demonstrated by Bassik *et al.* [35] pooled RNAi screens can be accurately analyzed by means of deep sequencing. Although this novel approach holds great promise for improved accuracy, the key issue of appropriate molecular tag selection remains unchanged. Due to the fact that the preparation of deep sequencing libraries involves a PCR step, the aforementioned problems resulting from self-annealing of hairpin sequences during amplification remain the same as for microarray analysis. Consequently, shRNA expression libraries with external barcodes can improve not only microarray but also deep sequencing analysis. A variety of exciting new applications for pooled RNAi screens will become possible with the increased sensitivity obtained from deep sequencing analyses, such as, for example, pooled genetic synthetic lethality screens [36, 37].

## SUMMARY

Microarray-based analysis methods for pooled RNAi screens in mammalian cell lines have rapidly developed over the past five years. Proof-of-concept has been provided and biologically relevant results, including identification of cancer cell essential genes [23-25], as well as potential drug target genes [21, 25], continue to be generated by means of such pooled screens. In this review, we summarize the approaches that have been taken so far to analyze pooled RNAi screens. We compare different molecular tags and point out the advantages of external barcode sequences over shRNA template related sequences. We further highlight the importance of appropriate target cell line selection before the commencement of pooled screens in order to achieve high knockdown efficiency. Since pooled RNAi screens in mammalian cells are currently only in the early stages, it will be exciting to watch their full potential develop.

## Figures and Tables

**Fig. (1):**
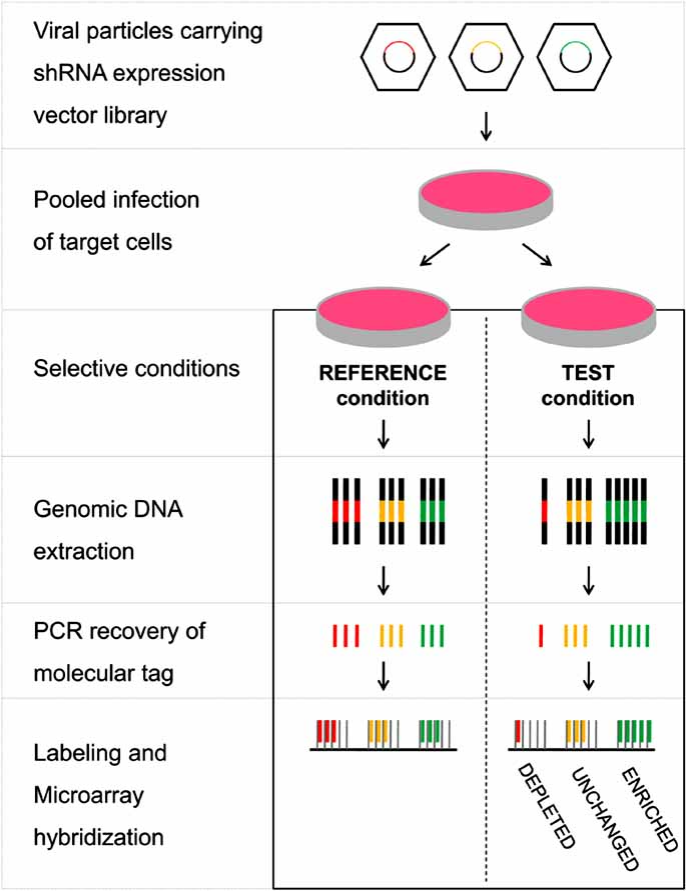
Schematic of the microarray-based analysis of a pooled RNAi screen.

**Table 1:** Overview of Selected Features from the Commercially Available shRNA Expression Libraries from the Hannon and Elledge Lab (H&E), the RNAi Consortium (TRC) and the Netherlands Cancer Institute (NKI). State of January 2010

	H&E library	TRC library	NKI library
**Distributor**	Open Biosystems, Thermo Scientific	Open Biosystems, Sigma-Aldrich	Geneservice
**Retroviral vector**	pSM2	none	pRS
**Lentiviral vector**	pGIPZ	pLKO1	none
**Number of covered genes**	18,000	15,000	8,000
**Avgerage constructs per gene**	2.5 (pGIPZ) 2.8 (pSM2)	5	3
**Total constructs**	45,000 (pGIPZ) 50,000 (pSM2)	80,000	24,000
**shRNA sequence**	22 nt stem, 19 nt loop	21 nt stem, 6 nt loop	19 nt stem, 9 nt loop
**Molecular tag**	half hairpin, external 60 nt barcode	half hairpin	full-length hairpin

## ABBREVIATIONS

RNAi: = Ribonucleic acid interference
shRNA: = Short hairpin RNA
siRNA: = Short interfering RNA
nt: = Nucleotide
H&E: = Hannon and Elledge
TRC: = The RNAi Consortium
NKI: = Nederlands Kanker Instituut
